# Diagnosing Alzheimer’s Disease Specifically and Sensitively With pLG72 and Cystine/Glutamate Antiporter *SLC7A11* AS Blood Biomarkers

**DOI:** 10.1093/ijnp/pyac053

**Published:** 2022-08-20

**Authors:** Hsien-Yuan Lane, Chieh-Hsin Lin

**Affiliations:** Department of Psychiatry and Brain Disease Research Center, China Medical University Hospital, Taichung, Taiwan; Graduate Institute of Biomedical Sciences, China Medical University, Taichung, Taiwan; Department of Psychology, College of Medical and Health Sciences, Asia University, Taichung, Taiwan; Graduate Institute of Biomedical Sciences, China Medical University, Taichung, Taiwan; Department of Psychiatry, Kaohsiung Chang Gung Memorial Hospital, Chang Gung University College of Medicine, Kaohsiung, Taiwan; School of Medicine, Chang Gung University, Taoyuan, Taiwan

**Keywords:** pLG72, *SLC7A11*, glutamate, N-methyl-D-aspartate, Alzheimer’s disease, biomarker

## Abstract

**Background:**

Reliable blood biomarkers for Alzheimer’s disease (AD) have been lacking. The D-amino acids oxidase modulator (named pLG72) modulates glutamate N-methyl-D-aspartate receptor activity. The cystine/glutamate antiporter contains a *SLC7A11* subunit, which mediates glutamate release. This study aimed to determine the accuracy of pLG72 protein and *SLC7A11* mRNA in diagnosing AD.

**Methods:**

This study enrolled 130 healthy controls and 109 unmatched AD patients; among them, 40 controls and 70 patients were selected to match by age. We measured their pLG72 protein in plasma and *SLC7A11* mRNA in white blood cells.

**Results:**

AD patients had markedly higher pLG72 levels and *SLC7A11* mRNA ΔCT values than healthy controls (in both unmatched and matched cohorts; all 4 *P* values <.001). The receiver operating characteristics analysis in the unmatched cohorts demonstrated that the pLG72 level had a high specificity (0.900) at the optimal cutoff value of 2.3285, the ΔCT of *SLC7A11* mRNA displayed an excellent sensitivity (0.954) at the cutoff of 12.185, and the combined value of pLG72 and *SLC7A11* ΔCT determined a favorable area under the curve (AUC) (0.882) at the cutoff of 21.721. The AUC of the combined value surpassed that of either biomarker. The specificity, sensitivity, and AUC of the matched cohort were like those of the unmatched cohort.

**Conclusions:**

The findings suggest that pLG72 protein and *SLC7A11* mRNA can distinguish AD patients from healthy controls with excellent specificity and sensitivity, respectively. The combination of pLG72 and *SLC7A11* yields better AUC than either, suggesting the superiority of simultaneously measuring both biomarkers in identifying AD patients.

**Significance Statement**

While blood biomarkers for Alzheimer’s disease (AD) have been lacking, this study enrolling 130 healthy controls and 109 AD patients found that AD patients had markedly higher pLG72 levels and *SLC7A11* mRNA ΔCT values than healthy controls. While *SLC7A11* mRNA levels showed better sensitivity than the pLG72 levels, the pLG72 levels revealed better specificity. Importantly, the combination of pLG72 and *SLC7A11* mRNA generated better AUC than either of the 2 biomarkers. Overall, the findings suggest that pLG72 protein joint with *SLC7A11* mRNA can constructively distinguish AD patients from healthy controls.

## Introduction

The prevalence of dementia in the elderly is increasing rapidly in the aging society, and the deteriorating clinical course is a heavy burden to both the patients and their family (Livingston et al., 2020). Early detection and intervention of Alzheimer’s disease (AD) is pivotal for the outcome (Budd et al., 2011), while, to date, the diagnosis of AD relies on medical history and behavioral observations (McKhann et al., 1984). The presence of characteristic neurological and neuropsychological features and the absence of other physical or mental conditions are supportive in diagnosis (Erkkinen et al., 2018). Advanced medical imaging with computed tomography (CT) or magnetic resonance imaging and with single-photon emission computed tomography or positron emission tomography can be used to help exclude other cerebral pathology or subtypes of dementia (Weiner et al., 2015).

Medical organizations have created diagnostic criteria to standardize the diagnostic procedure. For example, the National Institute of Neurological and Communicative Disorders and Stroke (NINCDS) and the Alzheimer’s Disease and Related Disorders Association (ADRDA, now known as the Alzheimer’s Association) established the most commonly used NINCDS-ADRDA criteria for diagnosis of AD (McKhann et al., 1984). However, the aforementioned diagnosis methods are time-consuming and dependent on the physicians’ experience. Therefore, it is necessary to search for blood biomarkers that can give rise to a quick, reliable, and accurate diagnosis of AD.

Currently, most studies of AD biomarkers in blood have mainly focused on known pathological substrates for the disease, such as amyloid plaques and neurofibrillary tangles, which are respectively composed of the abnormally aggregated amyloid-β peptide (Aβ) and hyperphosphorylated tau (Lewczuk et al., 2018, 2020). Recently, measuring the levels of t-tau/Aβ _42_, hyperphosphorylated tau_181_/Aβ _42_, and Aβ _42_/Aβ _40_ in blood samples successfully distinguished the AD patients from healthy controls (Kim et al., 2020).

However, the pathogenesis of AD remains still unresolved, and heterogeneous etiologies may be implicated (Lewczuk et al., 2020; Ahmed et al., 2021; Cheng et al., 2021; Tatulian, 2022). Optimal glutamate N-methyl-D-aspartate receptor (NMDAR) activation is required for synaptic plasticity, memory, and cognitive function (Tilleux and Hermans, 2007; Mattson, 2008). Attenuation of NMDAR-mediated neurotransmission can result in loss of neuronal plasticity and cognitive deficits in the aging brain, which may account for clinical deterioration and brain atrophy; on the other hand, overactivation of NMDAR leads to neurotoxicity (Madeira et al., 2018; Lin and Lane, 2019a; Pinheiro and Faustino, 2019; Segovia et al., 2001).

There are several avenues to enhance NMDAR activation and therefore cognitive function (Yao and Zhou, 2017; Cheng et al., 2021). One of them is inhibiting the activity of D-amino acids oxidase (DAAO), which is responsible for degrading D-amino acids such as D-serine (Fukui and Miyake, 1992; Vanoni et al., 1997; Sasabe et al., 2012; Pollegioni et al., 2018) and thereby raising D-serine levels and strengthening brain activity and cognitive function (Lin et al., 2014a; Yao and Zhou, 2017; Lane et al., 2021; Lin et al., 2021; Orzylowski et al., 2021; Ploux et al., 2021). The DAAO modulator (named pLG72) may affect the DAAO activity (Sacchi et al., 2016; Keller et al., 2018; Lin et al., 2020b) and play important roles in the modulation of NMDA signaling and in the pathogenesis of schizophrenia and AD (Lin et al., 2014b, 2019b, 2020b; Sacchi et al., 2016; Akyol et al., 2017; Keller et al., 2018).

Cystine/glutamate antiporter system x_c_^−^ has been also implicated in the pathogenesis of AD (Qin et al., 2006). System x_c_^−^, a sodium-independent acidic amino acid transporter, regulates the uptake of cystine into cells in exchange for glutamate in a 1:1 ratio (Bannai, 1986). System x_c_^−^ is composed of a light chain subunit (xCT, *SLC7A11*, which mediates the antiporter activity) and a heavy chain subunit (4F2hc, *SLC3A2*, which anchors the light chain subunit to the plasma membrane) (Bridges et al., 2012). Cystine is reduced to cysteine intracellularly after being taken up by system x_c_^−^. Cysteine is the rate-limiting substrate for the biosynthesis of glutathione, which is one of the most important antioxidants in the brain (Dringen and Hirrlinger, 2003). System x_c_^−^ also modulates glutamate release, while glutamate, the most abundant amino acid neurotransmitter in the mammalian brain (Tilleux and Hermans, 2007; Mattson, 2008), plays an important role in regulating cognitive aging (Yao and Zhou, 2017; Lin et al., 2019a; Chang et al., 2020). Patients with AD were found to have altered glutamate terminals in the hippocampus (Cowburn et al., 1988).

To our knowledge, the potential roles of pLG72 protein or cystine/glutamate antiporter *SLC7A11* in diagnosing AD have yet to be studied. To explore new blood biomarkers, this study aimed to determine the diagnostic accuracy of pLG72 protein and *SLC7A11* mRNA in detection of AD.

## Materials and Methods

### Participants

The study was approved by the Institutional Review Board of Chang Gung Memorial Hospital, Taiwan (104-9692B) and conducted in accordance with the current revision of the Declaration of Helsinki.

Both patients and healthy controls were evaluated by research psychiatrists after a thorough medical workup.

Patients were enrolled into this study if they (1) satisfied NINCDS-ADRD (McKhann et al., 1984) criteria for probable AD and had a clinical dementia rating (CDR) (Morris, 1993) score of ≥1, (2) were physically healthy and had all laboratory assessments (including blood routine and biochemical tests) within normal limits, and (3) agreed to participate in the study and provided informed consent.

Exclusion criteria included history of significant cerebrovascular disease with Hachinski Ischemic Score >4; major neurological, psychiatric, or medical conditions other than AD; substance (including alcohol) abuse or dependence; delusion, hallucination, or delirium symptoms; severe visual or hearing loss; and inability to follow protocol.

All healthy volunteers, aged ≥18 years, were free of any Axis I or II psychiatric disorder. To exclude potential confounding effects, all participants were non-smokers and had no DSM-IV diagnosis of substance (including alcohol) abuse. All healthy volunteers were physically and neurologically healthy and had normal laboratory assessments (including blood routine and biochemical tests).

### Laboratory Assessments

A total 10 mL of peripheral venous blood was collected into an ethylenediamin tetra-acetic acid tube by well-trained personnel. The specimens were immediately centrifuged at 1500×g for 10 minutes at 4°C for further assaying both pLG72 levels in plasma and *SLC7A11* mRNA levels in white blood cells (WBC).

#### Laboratory Methods for pLG72—

The laboratory methods for measuring pLG72 levels in plasma were previously detailed (Lin et al., 2019b). In brief, the blood specimens were centrifuged at 4°C and plasma was quickly dissected and immediately stored at −80°C until analysis. For western blotting, 100 µL plasma was depleted using ProteoPrep Blue Albumin and IgG Depletion Kit (Sigma, St. Louis, Missouri). The low-abundant protein fractions were collected to 100 μL, and 10 μL of them were mixed with 4× sample buffer (500 mM Tris-HCl [pH 6.8], 16% SDS, 80% glycerol, 400 mM DTT, and 0.08% bromophenol blue) and separated on 12% SDS-PAGE). Thereafter, proteins in the gels were transferred to 0.45 μm polyvinylidene difluoride membrane (Millipore), which was placed in 5% nonfat dry milk in TBST (20 mM Tris-HCl, pH 7.6, 500 mM sodium chloride, 0.1% Tween 20) for 1 hour at room temperature and then incubated with goat anti-pLG72 antibody (sc-46118, Santa Cruz Biotechnology) (Sacchi et al., 2008) diluted by 1:1000 in TBST overnight at 4°C. The membrane was washed thrice in TBST and incubated for 2 hours with an HRP-linked anti-goat IgG secondary antibody (sc-2030, Santa Cruz) diluted by 1:5000 in TBST. After 3 washes in TBST, the western blots were visualized with an ECL Advance Western Blotting Detection Kit (RPN2135, GE Healthcare). The stained membranes were photographed on ImageQuant LAS 4000 mini (GE Healthcare) and quantified using ImageQuant TL 7.0 software (GE Healthcare) by measuring the relative intensity from each band and normalized to the pLG72 recombinant protein (20 ng) signals. The commercial pLG72 antibodies were able to specifically recognize LG72 recombinant proteins. A standard curve was generated by serial dilutions of the pLG72 protein (50, 20, 10, 5, 2.5, 1.25, and 0.625 ng), and its detection limit was as low as 0.625 ng. The western blotting was repeated by 2 experienced technicians separately for quality control. The results of the blotting were very similar between the 2 technicians. The R-squared of the linearity between the blotting signals and the amounts of the pLG72 proteins was 0.988. In the western blotting, the molecular weight of the pLG72 protein band was approximately 18 kDa. The molecular weight of the standard recombinant pLG72 protein (as the control), which had a tagged protein on it, was marginally higher than that of the plasma pLG72 protein. The noise-signal ratios around the points of the western blotting were between 0.04 and 0.13.

#### Laboratory Methods for SLC7A11—

The laboratory procedure for determining the *SLC7A11* mRNA levels in WBC have been described elsewhere (Lin et al., 2016). In short, red blood cells were removed by 1× RBC lysis buffer (Genepure Technology Co., http://www.genepure.com.tw/index.asp), and WBC was obtained after blood centrifugation at 4°C. RNA was isolated from the WBC using the Tri-reagent method (MRC) according to the protocol provided by the manufacturer. The primer for *SLC7A11* was [CCATGAACGGTGGTGTGTT—GACCCTCTCGAGACGCAAC] located at No. 1269-1287 and No. 1329-1310 of the sequence (NM_014331.3, http://www.ncbi.nlm.nih.gov/nucleotide/80861465?report=genbank&log$=nuclalign&blast_rank=86&RID=XRBCU71A01R), and the mRNA expression of *SLC7A11* was measured by SYBR Green Master Mix on Real-Time PCR Detection System.

Four housekeeping genes were used as endogenous controls: glyceraldehyde-3-phosphate dehydrogenase NM_002046 and NM_002046.3, beta-2-microglobulin NM_004048.2, and hypoxanthine-guanine phosphoribosyltransferase NM_000194.2. The real-time quantitative PCR reaction was carried out twice for each sample. The relative mRNA level of *SLC7A11* in the samples was calculated by the ΔCt value (Ct_, target_ − Ct_, housekeeping_) (Livak and Schmittgen, 2001).

### Statistical Analysis

Baseline characteristics were calculated for the AD and control groups. Numeric data are presented as means ± SD. The *P* value between the AD and control groups was calculated based on *t* test or Fisher’s exact test, and *P *< .05 was considered to indicate statistical significance.

A receiver operating characteristics (ROC) analysis for pLG72 Protein, *SLC7A11* mRNA in ΔCT value, or the combination thereof was applied by plotting the proportion of true-positive results (sensitivity) vs the proportion of false-positive results (1 − specificity).

Logistic regression was applied to generate the predictive model for AD. In the model, AD (vs. control) was the dependent variable, and pLG72 protein level and SLC7A11 mRNA ΔCT value were the covariates. In this way, an equation (I) for the weighted value of pLG72 protein level and *SLC7A11* mRNA ΔCT value in combination as shown below was formulated using the logistic regression model:


$$\begin{array}{l} A=-13.246+B\times 3.197+C\times 2.273 \end{array}$$ (1)

A = Weighted value
B = pLG72 protein level (ng/μL)
C = ΔCT value of *SLC7A11* mRNA

## Results

### Participants

This study enrolled 239 participants, including 130 healthy controls and 109 unmatched AD patients. The mean age (74.6 ± 7.9 years [SD] years) of unmatched patients with AD was older than that of healthy controls (43.1 ± 18.2 years, *P* < .001).

Among them, we selected 70 patients from the AD group and 40 individuals from the healthy controls group to match by age.

The demographic and clinical data of age-matched and unmatched AD patients and healthy controls are summarized in Table 1.

**Table 1.** Demographic Characteristics of Patients With Dementia and Healthy Controls

	Unmatched		*P* ^*a*^	Matched with age		*P* ^*a*^
Parameter	Healthy controls	Patients with Alzheimer’s disease		Healthy controls	Patients with Alzheimer’s disease	
n	130	109		40	70	
Age (y)	43.1 ± 18.2	74.6 ± 7.9	<.001^*b*^	65.9 ± 13.0	70.1 ± 6.1	.059^*b*^
Gender, female (%)	63 (48.5)	71 (65.1)	.013^*b*^	17 (42.5)	47 (67.1)	.016^*c*^
Education (y)	13.5 ± 3.2	4.2 ± 4.1	<.001^*b*^	10.7 ± 3.4	4.4 ± 4.0	<.00^*b*^
BMI	28.5 ± 26.1	24.1 ± 3.9	.152^*b*^	24.6 ± 4.0	24.4 ± 3.8	.781^*b*^
CDR score	0	1.3 ± 0.6	<.001^*b*^	0	1.2 ± 0.5	<.001^*b*^
MMSE score	28.4 ± 1.2	16.8 ± 5.9	<.001^*b*^	28.4 ± 1.2	18.1 ± 5.7	<.001^*b*^
ADAS-cog score	5.1 ± 2.4	23.3 ± 11.7	<.001^*b*^	5.1 ± 2.4	21.3 ± 12.3	<.001^*b*^
Anti-dementia drug use	0	34	<.001^*c*^	0	24	<.001^*c*^
Donepezil	0	20		0	11	
Rivastigmine	0	5		0	5	
Galantamine	0	8		0	8	
Memantine	0	1		0	0	
pLG72 level (ng/μL)	1.47 ± 0.74	2.64 ± 1.17	<.001^*b*^	1.72 ± 0.71	2.63 ± 1.20	<.001^*b*^
Anti-dementia drug group		2.22 ± 1.04			2.06 ± 1.03	
Drug free group		2.84 ± 1.18			2.93 ± 1.18	
mRNA of *SLC7A11*^*d*^	12.10 ± 1.65	13.89 ± 1.21	<.001^*b*^	12.44 ± 1.37	13.82 ± 1.29	<.001^*b*^
Anti-dementia drug group		13.55 ± 1.04			13.54 ± 1.14	
Drug-free group		14.05 ± 1.25			13.97 ± 1.32	

Abbreviations: ADAS-cog, The Alzheimer’s Disease Assessment Scale–Cognitive Subscale; BMI, body mass index; CDR, Clinical Dementia Rating Scale; MMSE, Mini-Mental State Examination.

^*a*^ *P* value between healthy controls and patients with dementia.

^*b*^ *t* test.

^*c*^Fisher’s exact test.

^*d*^Delta CT values of mRNA expressions of *SLC7A11*.

Among the 109 unmatched patients with AD, 34 were medicated and the other 75 were drug free. Among the 70 matched patients with AD, 24 were medicated and the other 46 were drug free (Table 1).

### pLG72 Protein

**Figure 1.**
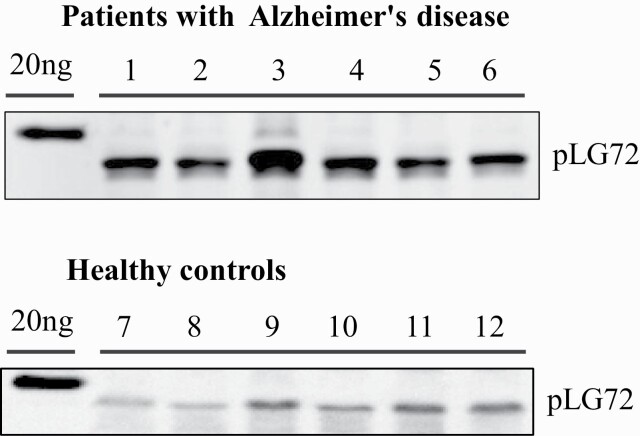
Western blotting results of pLG72 protein in plasma from randomly selected patients with Alzheimer’s disease (n = 6) and healthy controls (n = 6).

Typical western-blot results for G72 protein in plasma samples from randomly selected AD patients (n = 6) and healthy controls (n = 6) are shown in Figure 1.

As shown in Table 1, the pLG72 protein levels in the plasma of unmatched and matched AD patients were markedly higher than those of healthy controls.

The mean levels (SD) of pLG72 protein in unmatched AD and healthy controls were 2.64 ± 1.17 ng/μL and 1.47 ± 0.74 ng/μL, respectively (*P* < .001). The medicated patients had lower pLG72 levels than the drug-free patients (2.22 ± 1.04 ng/μL vs 2.84 ± 1.18 ng/μL; *P* = .01, Mann-Whitney U test).

The mean levels (SD) of pLG72 protein in matched AD and healthy controls were 2.63 ± 1.20 ng/μL and 1.72 ± 0.71 ng/μL, respectively (Table 1; *P* < .001). The medicated patients had lower pLG72 levels than the drug-free patients (2.06 ± 1.03 ng/μL vs 2.93 ± 1.18 ng/μL; *P *=* *.003, Mann-Whitney U test) (Table 1).

### SLC7A11 mRNA

Also shown in Table 1, the ΔCT values of *SLC7A11* mRNA in WBC of healthy controls were markedly lower than those of AD patients.

The mean expression levels (SD) of the ΔCT values of *SLC7A11* mRNA in unmatched AD and healthy controls were 13.89 ± 1.21 and 12.10 ± 1.65, respectively (*P* < .001). The medicated patients had higher mRNA of *SLC7A11* (ΔCT value, 13.55 ± 1.04 ng/μL vs 14.05 ± 1.25 ng/μL; *P *=* *.046, *t* test) (Table 1).

The mean expression levels (SD) of the ΔCT values of *SLC7A11* mRNA in matched AD and healthy controls were 13.82 ± 1.29 and 12.44 ± 1.37, respectively (Table 1; *P* < .001). The medicated patients and the drug-free patients had similar mRNA of *SLC7A11* (ΔCT value, 13.54 ± 1.14 ng/μL vs 13.97 ± 1.32 ng/μL; *P* = .174, *t* test) (Table 1).

### ROC Analysis for Specificity and Sensitivity

**Figure 2.**
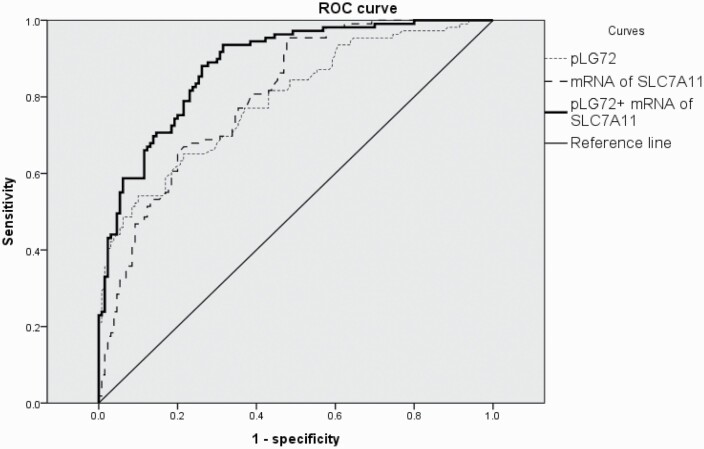
ROC curves of plasma pLG72 protein level and mRNA of *SLC7A11* in white blood cells of all healthy controls vs all patients with Alzheimer’s disease.

Shown in Table 2 and Figure 2, ROC analysis was applied to determine the cutoff values of pLG72 protein and *SLC7A11* mRNA as potential AD predictors by plotting the proportion of true-positive results (sensitivity) vs the proportion of false-positive results (1 − specificity).

**Table 2.** ROC Curve Analysis and Multivariate Logistic Regression of Plasma pLG72 Protein Level and mRNA of *SLC7A11* in White Blood Cells of Healthy Controls vs Patients With Alzheimer’s Disease

	ROC curve analysis			
	Cut-off	Sensitivity	Specificity	AUC
pLG72				
Unmatched AD	>2.3285	54.1	90.0	0.791
Matched AD	>2.3285	54.3	90.0	0.726
Unmatched drug-free AD	>2.3285	64.0	90.0	0.829
mRNA of *SLC7A11*^*a*^				
Unmatched AD	>12.185	95.4	52.3	0.803
Matched AD	>12.185	92.9	45.0	0.764
Unmatched drug-free AD	>12.185	96.0	52.3	0.824
pLG72+ mRNA of *SLC7A11*^*a*^,^*b*^				
Unmatched AD	>21.723	88.1	73.8	0.882
Matched AD	>21.723	85.7	67.5	0.833
Unmatched drug-free AD^*c*^	>24.985	90.7	77.7	0.915

Abbreviations: AD, Alzheimer’s disease; AUC, area under the curve.

^*a*^Delta CT values of mRNA expressions of *SLC7A11*.

^*b*^An equation calculated by using the logistic regression model with pLG72 and mRNA expressions of *SLC7A11* as the covariates (−13.246+G72*3.197+ mRNA expressions of SLC7A11*2.273).

^*c*^An equation calculated by using the logistic regression model with pLG72 and mRNA expressions of *SLC7A11* as the covariates in unmatched drug-free AD patients (−15.870+G72*4.034+ mRNA expressions of SLC7A11*2.553).

The ROC analysis of the pLG72 levels for all AD patients vs healthy controls determined an optimal cutoff value (2.3285) with a modest sensitivity (0.541) and a high specificity (0.900) (AUC = 0.791) (Table 2). The ROC analysis of the ΔCT values of *SLC7A11* mRNA for all AD patients vs healthy controls determined an optimal cutoff value (12.185) with an excellent sensitivity (0.954) and a modest specificity (0.523) (area under the curve [AUC] = 0.803) (Table 2).

The ROC analysis of the combined values of pLG72 levels and the ΔCT values of *SLC7A11* mRNA for all AD patients vs healthy controls determined an optimal cutoff value (21.721) with a favorable sensitivity (0.881) and a modest specificity (0.738) (AUC = 0.882) (Table 2; Figure 2). The AUC of the combined values of the 2 biomarkers was better than the AUC of either of the 2 biomarkers (Table 2; Figure 2).

The specificity, sensitivity, and AUC of the matched AD patients and healthy controls were similar to those of the unmatched cohort (Table 2). ROC curves of pLG72 protein and mRNA of *SLC7A11* of matched healthy controls vs AD patients are not shown because they are similar to Figure 2.

Among the drug-free patients, the ROC analysis of the combined values of pLG72 levels and the ΔCT values of *SLC7A11* mRNA for AD patients vs healthy controls determined an optimal cutoff value (24.985) with a favorable sensitivity (0.907) and a modest specificity (0.777) (AUC = 0.915). The AUC of the combined values of the 2 biomarkers were better than the AUC of either of the 2 biomarkers (0.829 and 0.824, respectively) (Table 2).

From the above results, the differentiating power of the combined values of pLG72 levels and the ΔCT values of *SLC7A11* mRNA was better in the drug-free AD patients than the medicated.

### Correlations Between Levels of pLG72 and *SLC7A11* mRNA and Scores of CDR, MMSE, and ADAS-cog

Among the AD patients, we checked the correlations between levels of pLG72 and *SLC7A11* mRNA and scores of CDR, MMSE, and ADAS-cog. There was no correlation between levels of pLG72 and scores of CDR (r = −0.037, *P* = .703), MMSE (r = −0.116, *P *=* *.247), and ADAS (r = −0.024, *P *=* *.808). There was also no correlation between levels of SLC7A11 and scores of CDR (r = 0.175, *P *=* *.069), MMSE (r = −0.164, *P *=* *.100), and ADAS (r = 0.091, *P *=* *.361).

Among the healthy controls, we checked the correlations between levels of pLG72 and *SLC7A11* mRNA and scores of MMSE and ADAS-cog (but not CDR), because all CDR scores of healthy controls were zero. There was no correlation between levels of pLG72 and scores of MMSE (r = −0.126, *P *=* *.514) and ADAS (r = 0.030, *P *=* *.876). There was also no correlation between levels of SLC7A11 and scores of MMSE (r = −0.214, *P *=* *.265) and ADAS (r = 0.265, *P *=* *.164).

## Discussion

The main findings of this study suggest that while pLG72 (DAOA) protein or *SLC7A11* mRNA alone may be useful as a potential biomarker for the detection of AD, the combination of pLG72 protein and *SLC7A11* mRNA can lead to an even more reliable diagnosis of AD.

pLG72, existing in mitochondria of humans and the other 3 primate species (Chumakov et al., 2002), has been reported to correlate with DAAO (Mechelli et al., 2012; Birolo et al., 2016; Keller et al., 2018; Lin et al., 2020b). DAAO levels in serum have been found to be increased with the severity of the cognitive aging; however, DAAO blood concentration has been inadequate to be a good enough biomarker for AD (Lin et al., 2017). The current study indicates that pLG72 levels in plasma display a high specificity (0.900) in differentiating AD patients from healthy controls.

Cystine/glutamate antiporter system x_c_^−^ also has been implicated in the pathogenesis of not only AD (Qin et al., 2006) but also schizophrenia (Fournier et al., 2017). In fact, there are overlapping pathophysiology and clinical manifestations between AD and schizophrenia: both reveal cognitive decline (McKhann et al., 1984; Lin et al., 2014c), psychotic and behavioral symptoms (Lin and Lane, 2019a; Kendler, 2020), implication with NMDAR pathogenesis (Yao and Zhou, 2017; Lin and Lane, 2019b), and clinical improvement by DAAO inhibition (Harrison, 2018; Lin et al., 2020a). Previous study (Lin et al., 2016) has suggested that the mRNA expression of cystine/glutamate antiporter system x_c_^−^*SLC3A2* may have potential to be a biomarker of schizophrenia in the future. The present study demonstrated that cystine/glutamate antiporter *SLC7A11* mRNA had supersensitivity for identification AD.

Of note, the combination of pLG72 and *SLC7A11* generated higher AUC than either biomarker, therefore suggesting the superiority of the combination in diagnosing AD. Since both pLG72 and cystine/glutamate antiporter system x_c_^−^ mediate glutamate-related neurotransmission and redox reaction (Filiou et al., 2012; Fournier et al., 2017; Lin and Lane, 2019b), it is reasonable for simultaneously measuring both pLG72 and *SLC7A11*.

We compared the 2 biomarkers in age-unbalanced and age-balanced cohorts, mainly to examine whether age may affect the biomarker-based differentiation between the AD patients and healthy controls. We found that the results in the age-unbalanced cohorts and age-balanced cohorts were quite similar, implying that age may not confound the differentiation.

The present study has several limitations. First, the findings of this study were obtained from a cross-sectional design. Future longitudinal studies are warranted. Second, the blood-brain (or CSF) relationship of pLG72 and *SLC7A11* remain uncertain in the participants. Third, we did not apply mass spectrometry to confirm the recognized band in western blotting corresponding to pLG72. Fourth, only Han Chinese populations were recruited in this study. Whether the findings can be extrapolated to other populations deserves investigation. Fifth, the sample size in the matched cohort was modest. Sixth, western-blot analyses were not done in triplicate. Seventh, we did not measure biomarkers of AD pathophysiology (Aβ or tau) for confirmation. Eighth, APOE4 was not assayed. And lastly, there was an almost significant *P* value for the age-matched cohort in terms of age; in addition, the control group and the AD group differed in gender distribution and education levels. Since women are more likely to have AD (Prince et al., 2016) and low education is an important risk factor for dementia (Maccora et al., 2020), we regard the difference in gender (female predominance) and education in the study as nature. Further study on participants with better matched age, gender distribution, and education levels is necessary.

In conclusion, the above findings suggest that the pLG72 protein levels and *SLC7A11* mRNA levels (as expressed in ΔCT values) can differentiate AD patients and healthy controls with good AUC. *SLC7A11* mRNA levels have better sensitivity than pLG72 levels, while pLG72 levels have better specificity. The more interesting finding is that the combination of pLG72 and SLC7A11 yields better AUC than either, suggesting the superiority of simultaneously measuring both biomarkers in identifying AD patients.
